# Commentary: Understanding the Impact of Infection, Inflammation and Their Persistence in the Pathogenesis of Bronchopulmonary Dysplasia

**DOI:** 10.3389/fmed.2017.00024

**Published:** 2017-03-02

**Authors:** Jay K. Kolls

**Affiliations:** ^1^Department of Pediatrics, Richard King Mellon Institute for Pediatric Research, University of Pittsburgh School of Medicine, Pittsburgh, PA, USA

**Keywords:** bronchopulmonary dysplasia, IL-1, CXCL8, CXCR2, lung diseases

As Drs. Balany and Bhandari (1) point out in their recent review, despite the development of surfactant therapy, and new forms of non-invasive ventilation, bronchopulmonary dysplasia (BPD) remains the most common chronic respiratory disease in newborns/infants. The authors point out the key drivers of disease including infection, barotrauma, hyperoxia, and inflammation and highlight the potential role that inflammation plays in driving the disease. Lung inflammation can be triggered by activation of the innate immune system and this can be triggered by cell surface or cytoplasmic receptors that fall into the three key categories—toll-like receptors (TLRs), Nod-like receptors (NLRs), and c-type lectin receptors (CLRs). TLRs 1, 2, 4, and 6 are surface receptors that recognize both exogenous stimuli such as lipopeptides or endotoxin (2) and endogenous ligands such as Hsp70 or oxidized phospholipids (3). Thus, inflammation in BPD could be driven by ligands from invading pathogens in an intubated patient or endogenous ligands that could be released during hyperoxia. One could envision that the therapeutic approach may be different given the proximal driver of inflammation. Thus, it is important to not only to characterize the type of inflammation but we need a keen understanding of what initiated the response. NLRs are cytoplasmic receptors and have several ligands including muramyl dipeptide (2). These receptors play a key role in activation of the inflammasome which is an intracellular protein structure that results in caspase-1 mediated cleavage of proIL-1β or pro-IL-18 into mature secreted proteins. By contrast, other IL-1 family members such as IL-1α or IL-33 can be released and be biologically active independent of caspase-1-mediated cleavage. This pathway is an active area of drug development as there is strong genetic evidence of IL-33 in asthma (4, 5). For ligands that bind IL-1R1, IL-1β, and IL-1α, Anakinra has been FDA approved for the treatment of rheumatoid arthritis and for the treatment of neonatal-onset multisystem inflammatory disease (5). Given that overexpression of IL-1β results in decreased alveolarization (6), this may be a viable target. However, this pathway is also critical for host immunity to several pathogens including influenza (7) and *Staphylococcus aureus* infection (8), so one would need to proceed with caution and likely exclude patients with active infection. CLRs recognize carbohydrate ligands such as mannans and glucans and drive inflammation but also can be involved in resolution of inflammation as well (9).

As Drs. Balany and Bhandari state in their review (1), cytokines have been extensively evaluated in BPD including IL-1, IL-6, TNF, and IL-10. Moreover the CXCR2 ligands, CXCL1, and CXCL8 have also been found to be elevated in BPD. In preclinical models, CXCR2 antagonism appears to improve lung histology (10). CXCR2 antagonists have been studied in human clinical trials in cystic fibrosis and COPD. A recent phase 2 study showed an increased in FEV1 in COPD (11), and another trial showed reduced inflammation in sputum biomarkers in CF (12). Thus, this may be an approach in BPD. In the adult lung, CXCL8 can be made by both alveolar and tissue macrophages as well as the lung epithelium. It has been recently show in neonates that γδ T-cells are a major source of CXCL8 (13) and thus as the sources of CXCL8 may be unique in the neonatal lung—this will need to be taken into consideration for rational drug approaches.

Finally is the issue of resolution of inflammation. Namely, is BPD due to a failure to resolve inflammation? To this end, a critical area of needed research in this regard is the role of lipid mediators such as resolvins and lipoxins in BPD (14). Lipoxin A4 has been shown to be reduced in chronic neutrophilic lung inflammation in CF (15). Thus, in addition to cytokine measurement, there needs to be more research in assaying both pro-inflammatory and pro-resolving lipid mediators in the lungs of BPD subjects to determine if this may also be a target for therapeutic intervention. Moreover, we need more basic understanding of how gestational age and lung maturity affect the inflammatory response in the lung—both in terms of intitiating a response and in it’s resolution.

In conclusion, BPD is clearly a disease associated with airway inflammation with likely a complex series of initiators. However, defining inflammation—using unbiased omic approaches (proteomics and lipidomics) as well as replication studies in well-characterized patient cohorts may improve our understanding of the potential role of inflammation in disease pathogenesis and open up new avenues of therapeutic intervention.

## Author Contributions

The author confirms being the sole contributor of this work and approved it for publication.

## Conflict of Interest Statement

The author declares that the research was conducted in the absence of any commercial or financial relationships that could be construed as a potential conflict of interest.
